# Uttroside B, a US FDA-designated ‘Orphan Drug’, mitigates the development of hepatocellular carcinoma and its pulmonary metastasis via EGFR/ERK-mediated inhibition of SREBP-1 and STAT-3

**DOI:** 10.1038/s41420-026-03055-5

**Published:** 2026-04-16

**Authors:** Chenicheri Kizhakkeveettil Keerthana, Tennyson P. Rayginia, Kalishwaralal Kalimuthu, Sandhini Saha, Nair H. Haritha, Amrutha Nisthul Areekkara, Mundanattu Swetha, Sreekumar U. Aiswarya, Lekshmi R. Nath, Sanjay Suresh Varma, Arun Viswanathan, Jannet S, Shirly J, Aparna JS, Archana Praveen, Vishnu Sunil Jaikumar, Sankar Sundaram, Nikhil Ponnoor Anto, Tushar K. Maiti, C Sadasivan, Noah Isakov, Ravi S. Lankalapalli, Kuzhuvelil B. Harikumar, Ruby John Anto

**Affiliations:** 1 Division of Cancer Research, BRIC-Rajiv Gandhi Centre for Biotechnology, Thiruvananthapuram, India; 2 Department of Biotechnology, University of Kerala, Thiruvananthapuram, India; 3 Molecular Bioassay Laboratory, Institute of Advanced Virology, Thiruvananthapuram, India; 4 Regional Centre for Biotechnology, Faridabad, India; 5 Department of Biotechnology and Microbiology, Thalassery Campus, Kannur University, Kannur, India; 6 Department of Pharmacognosy, Amrita School of Pharmacy, Amrita Vishwa Vidyapeetham, AIMS Health Science Campus, Kochi, India; 7 Chemical Sciences and Technology Division, CSIR-National Institute for Interdisciplinary Science and Technology, Thiruvananthapuram, India; 8 Department of Pathology, Government Medical College, Kottayam, India; 9 The Shraga Segal Department of Microbiology, Immunology and Genetics, Faculty of Health Sciences, Ben-Gurion University of the Negev, Beer Sheva, Israel; 10 Centre of Excellence in Nutraceuticals, KSCSTE, Government of Kerala, Thiruvananthapuram, India

**Keywords:** Cancer, Cancer prevention

## Abstract

Hepatocellular carcinoma (HCC) is a highly aggressive tumor with rapid propensity for extrahepatic metastasis, which critically limits long-term clinical benefits of conventional chemotherapeutics and decreases the overall survival rate of patients. Our previous reports have documented the anti-HCC potential and pharmacological safety of uttroside B (Utt-B). Herein, we illustrate the role of EGFR/ERK signaling axis and their downstream targets SREBP-1 and STAT-3, in the action mechanism of Utt-B. Further, the current study also demonstrates the strong anti-invasive and anti-metastatic properties of Utt-B against liver cancer. Pharmacological inhibition of EGFR/ERK axis led to the abrogation of Utt-B-mediated cytotoxicity and induction of apoptosis, in vitro. siRNA-mediated silencing of EGFR resulted in the attenuation of the cytotoxic, pro-apoptotic and anti-invasive effects of Utt-B, in vitro, thereby validating the regulatory role of EGFR in orchestrating the anti-HCC and anti-metastatic potential of Utt-B. In vivo studies confirmed that treatment with Utt-B mitigates the development of primary hepatic tumors in an orthotopic xenograft model and impedes the pulmonary metastasis of HCC in a murine metastasis model, via the down-regulation of EGFR/ERK axis. Taken together, the current findings attest to the exceptional therapeutic potential of Utt-B against primary and metastatic HCC and highlight its potential as a candidate drug to be evaluated in the clinics for the benefit of HCC patients having limited prognosis and therapeutic options.

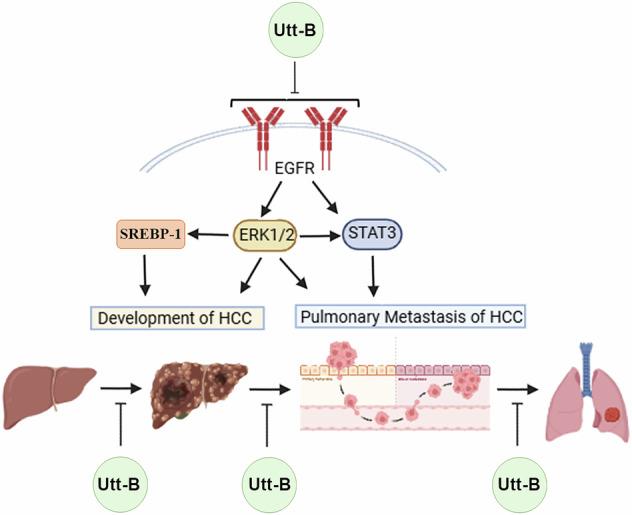

## Introduction

Hepatocellular carcinoma (HCC) accounts for about 90% of malignant liver cancer and is the third leading cause of cancer-related mortality, worldwide [1, 2]. In spite of the significant progress in the therapeutic options for HCC over the past decade, the clinical outcome in terms of overall survival rates of patients with advanced HCC is still unsatisfactory, owing to its high recurrence rates [3] and high propensity of the tumors to directly invade the portal and hepatic veins. Pulmonary metastasis constitutes around 49% of extrahepatic hematogenous metastasis in HCC patients [4, 5]. The prognosis and treatment outcomes in advanced HCC cases with pulmonary metastasis are poor. EGFR over-expression has been reported in over 60% of primary HCC cases [6]. Major survival signaling cascades such as the Ras/Raf/MEK/ERK, JAK/STAT and the PI3K/Akt/mTOR pathway occur downstream of activated EGFR signaling [7]. Interestingly, ERK, a down-stream target of EGFR, is known to activate SREBP-1, the key transcription factor governing various rate-limiting enzymes involved in lipid metabolism [8, 9]. Previous literature suggests that high expression of SREBP-1 leads to excessive de novo lipogenesis and lipid uptake which in turn drive the initiation, malignant transformation and progression of HCC [10]. Notably, STAT3, non-canonically activated by receptor tyrosine kinases such as EGFR, plays a pivotal role in epithelial- to-mesenchymal transition and metastatic progression of HCC [11, 12].

Previous studies conducted in our lab have enumerated the anti-HCC effect of Utt-B and pharmacological safety of the compound [13]. Notably, our findings on the exceptional therapeutic efficacy of Utt-B against HCC have been granted patents from the USA (US20190160088), Europe (EP3463382), Canada (3,026,426), South Korea (KR1020190008323) and Japan (JP2019520425). Our studies suggest that Utt-B exhibits superior anti-cancer properties against HCC compared to sorafenib, the US FDA-approved first-line anti-HCC drug [14]. Furthermore, we have previously reported the therapeutic efficacy and pharmacological safety of a novel combinatorial regimen involving Utt-B and sorafenib for combating HCC [15]. Our previous findings suggest that Utt-B promotes pro-survival autophagy in hepatic cancer and blockade of Utt-B-mediated autophagic response enhances the pro-apoptotic function of the compound against HCC [16]. Although it is known that Utt-B induces down-regulation of MAPK and mTOR signaling, the key regulator controlling the therapeutic effects of Utt-B has not yet been identified. Moreover, the influence of Utt-B on the metastatic progression of HCC has not been explored yet. Hence, the present study aims to elucidate the pharmacodynamics of Utt-B and evaluate its anti-metastatic potential in the context of HCC.

## Results

### Uttroside B exhibits anti-HCC effects by targeting EGFR, mTOR and MAPK pathways

**Fig. 1:**
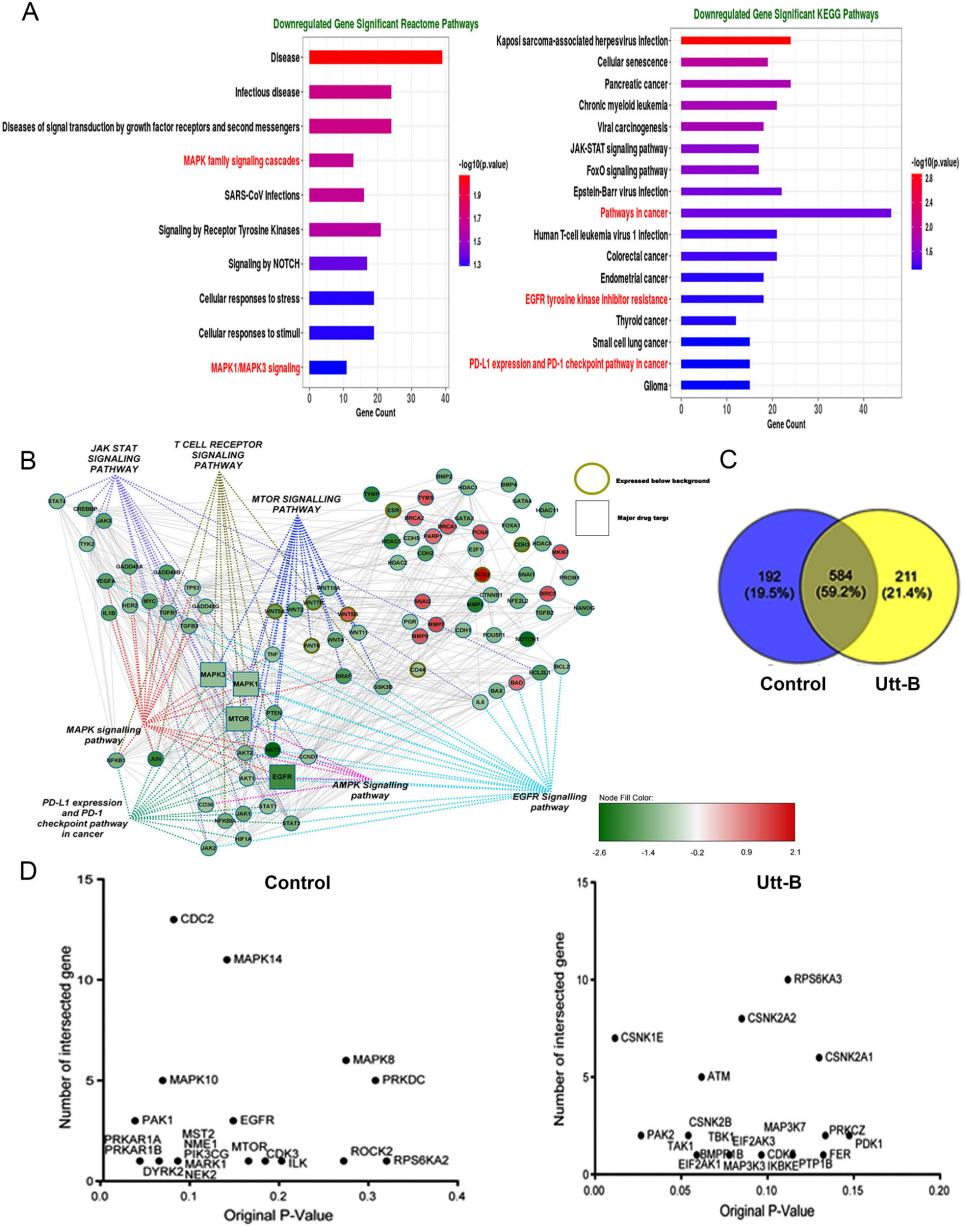
Transcriptomic and phosphoproteomic analyses reveal that EGFR, MAPK and mTOR pathways are the major targets of Utt-B. **A** Pathway analysis of Nanostring nCounter assay data using KEGG and Reactome databases depicts the list of significantly down-regulated pathways in HepG2 cells treated with Utt-B, compared to control. **B** STRING analysis showing direct interactions of the down-regulated genes with major signaling pathways associated with the progression of HCC and induction of anti-tumor immune response. **C** Phosphoproteomics analysis depicting the number of differentially expressed phosphoproteins in the control and Utt-B-treated HepG2 cells. **D** Kinome extraction data showing the enriched kinases unique to the control and Utt-B treatment groups.

**Fig. 2:**
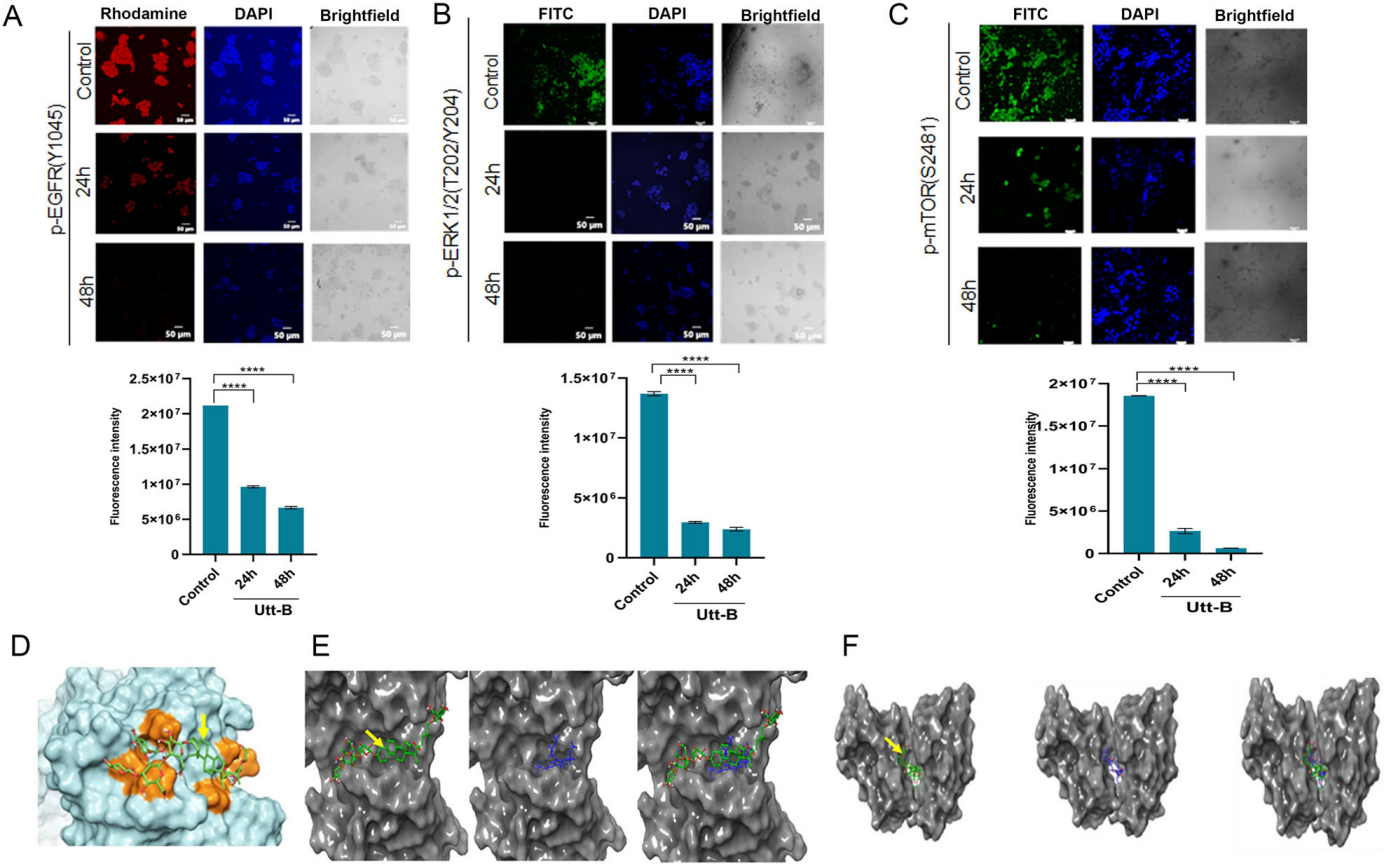
In vitro and in silico studies indicate the inhibitory effects of Utt-B on EGFR, ERK and mTOR. Immunocytochemical analysis showing the time-dependent changes in the phosphorylation of **A** p-EGFR, **B** p-ERK1/2 and **C** p-mTOR in HepG2 cells. One-way ANOVA followed by Tukey’s post hoc t test analysis was used for statistical comparison between different groups for experiments. ****P ≤ 0.0001. **D** Docking pose of Utt-B with the extracellular domain of EGFR as analyzed by in silico studies. Utt-B (indicated in green color and pointed by yellow arrow) binds at the inhibitory site in the extracellular domain of EGFR and forms three H-bonds with Q384, S418 and K465 residues (indicated in orange color). **E** Docking pose and fitness of Utt-B with the kinase domain of EGFR as analyzed by in silico studies. From left, docking pose and orientation of Utt-B (indicated in green color and pointed by yellow arrow) binding at the inhibitory site in the kinase domain of EGFR, docking pose and orientation of erlotinib indicated in blue color) with EGFR and comparative docking pose and orientation of erlotinib and Utt-B with EGFR, depicting that Utt-B binds at the same site as that of erlotinib. **F** Docking pose and fitness of Utt-B with the kinase domain of mTOR as analyzed by in silico studies. From left, docking pose and orientation of Utt-B (indicated in green color and pointed by yellow arrow) binding at the inhibitory site in the kinase domain of mTOR, docking pose and orientation of rapamycin indicated in blue color) with mTOR, and comparative docking pose and orientation of rapamycin and Utt-B with mTOR, depicting that Utt-B binds at the same site as that of rapamycin.

Utt-B-mediated changes at the transcriptome level were assessed by performing a Nanostring nCounter assay using HepG2 cells treated with the IC50 concentration of the compound i.e.,500 nM. The results of the nCounter assay revealed that Utt-B significantly lowers the expression of several genes associated with HCC progression, including, EGFR, STAT3, VEGF, MYC, BRAF, TGF-β1, JAK1, JUN, HIF-1α, CTNNB1 and FOXA1. Interestingly, Utt-B treatment also induced a marked decrease in the expression of CDH2, SNAI1 and MMP3, the most prominent molecular markers associated with cancer metastasis (Supplementary Fig. 1A). The functional analysis and STRING analysis of the Nanostring dataset using KEGG and Reactome pathway databases revealed that EGFR, MAPK and JAK/STAT pathways are the major pathways being down-regulated in response to Utt-B (Fig. 1A, B). The phosphoproteomic profiling of the control and Utt-B-treated HepG2 cells revealed that a total of 192 unique phosphoproteins were expressed in the control cells, and 211 phosphoproteins were expressed in cells treated with Utt-B, while a total of 584 phosphoproteins were commonly expressed in both groups (Fig. 1C; Supplementary Fig. 1B, C, Supplementary Data file). Further, a kinome extraction of the phosphoproteomics dataset revealed that the major kinases enriched in the control condition include EGFR, MAPK and mTOR. The expression of PAK1, an inducer of the MAPK survival signaling cascade [17], was observed in the control condition. On the contrary, there was paradigm shift in the enriched kinases in the cells treated with Utt-B. EGFR, mTOR and major MAPK kinases were absent in the treated condition. Most of the enriched kinases in the Utt B-treated group were pro-apoptotic kinases. Interestingly, the expression of TAK1, a prominent kinase involved in the transient phosphorylation and endocytosis of the EGFR [18], was observed in the Utt-B treated samples. Moreover, neither EGFR, nor mTOR and MAPK were present in the treated condition (Fig. 1D). Thus, the results of the phosphoproteomics analysis suggest that the major molecular targets of Utt-B are, EGFR, mTOR and MAPK. Immunocytochemical analysis revealed that Utt-B induced a time-dependent decrease in the expression of p-EGFR (Y1045), p-mTOR (S2481) and p-ERK1/2 (T202/Y204) in HepG2 cells (Fig. 2A–C). In silico molecular docking analyses were performed to decipher the possible direct physical interaction of the compound with any of its molecular targets. When the compound was docked with EGFR, it was observed that Utt-B could bind in the extracellular domain of EGFR at the same site, where cetuximab, the anti-EGFR monoclonal antibody binds (Fig. 2D). The glide gscore value of Utt-B with EGFR is −7.41 kcal/mol. Interestingly, Utt-B also exhibited binding feasibility in the kinase domain of EGFR, at the same site where erlotinib, the pharmacological inhibitor of EGFR binds (Fig. 2E). The glide gscore value of Utt-B is −9.77 kcal/mol, which is slightly more negative than the gscore of erlotinib (−9.25 kcal/mol), indicating better binding affinity and stability of interaction between Utt-B and EGFR, over erlotinib. Further, the docking studies of Utt-B with mTOR revealed that Utt-B could bind at the same inhibitory site in the kinase domain of mTOR, where the pharmacological inhibitor of mTOR, rapamycin binds (Fig. 2F). The glide gscore value of Utt-B is −8.05 kcal/mol, which is far greater than that of rapamycin (−15.9 kcal/mol), indicating the better binding affinity and stability of interaction between rapamycin and mTOR, over Utt-B. Hence, the in silico findings suggest the possibility of direct physical interaction of Utt-B with EGFR rather than mTOR, owing to the better docking fitness.

### Pharmacological inhibition of EGFR/ERK axis abrogates the anti-HCC potential of uttroside B, attesting the regulatory role of this signaling axis

**Fig. 3:**
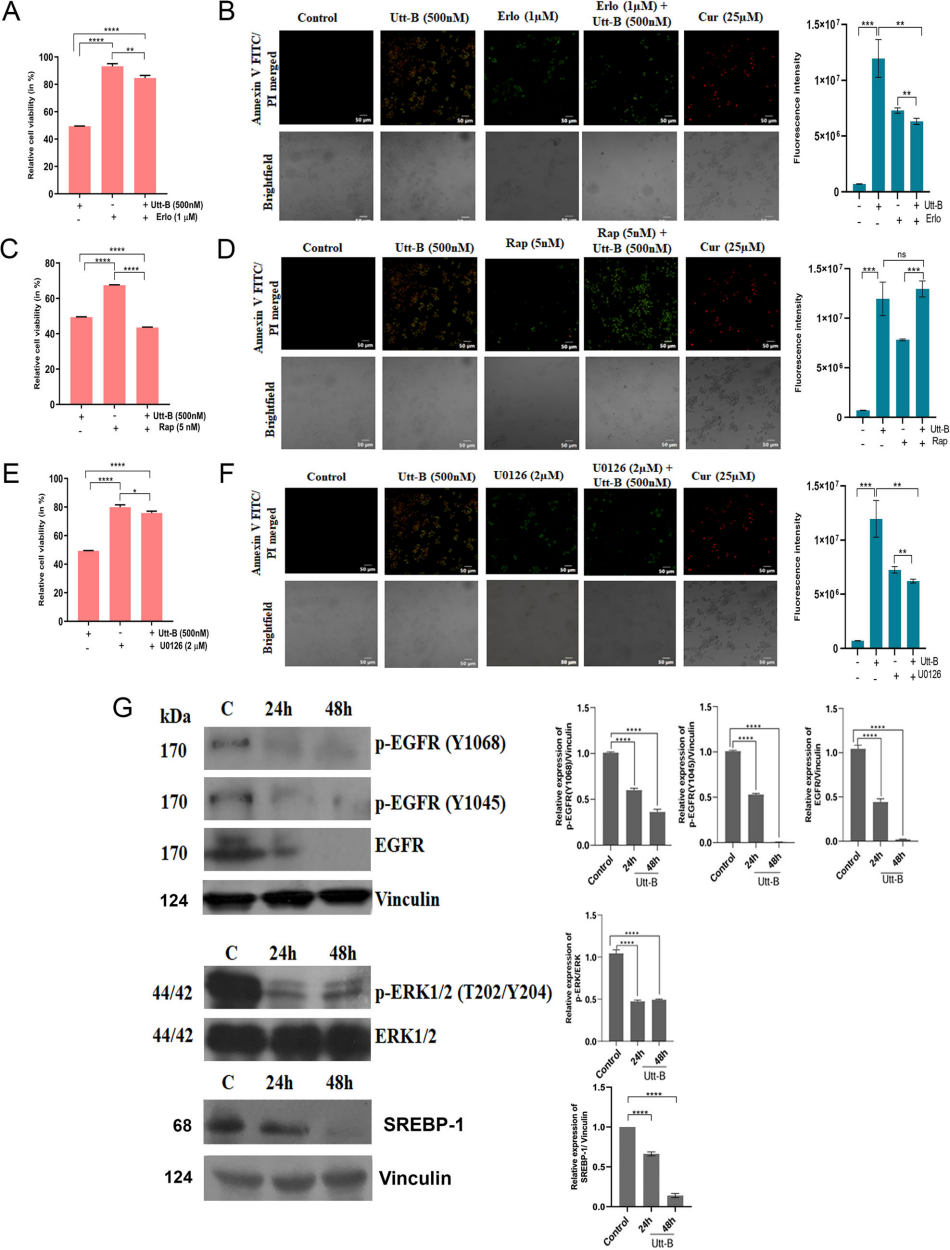
Pharmacological inhibition of EGFR/ERK axis abrogates the therapeutic effects of Utt-B against HCC. MTT assay depicting the changes in Utt-B-induced cytotoxicity in HepG2 cells upon pharmacological inhibition of **A** EGFR with erlotinib, **C** mTOR with rapamycin and **E** MAPK/ERK with U0126. One-way ANOVA followed by Tukey’s post hoc t test analysis was used for statistical comparison between different groups. ****P ≤ 0.0001; **P ≤ 0.01; *P ≤ 0.05; ns- non-significant. Annexin V/PI staining showing the changes in Utt-B-mediated induction of apoptosis in HepG2 cells upon pharmacological inhibition of **B** EGFR with erlotinib, **D** mTOR with rapamycin and **F** MAPK/ERK with U0126. Student's t test analysis was used for statistical comparison between different groups. ***P ≤ 0.001; **P ≤ 0.01; ns- non-significant. **G** Western blot showing the down-regulation of EGFR/ERK axis and SREBP-1 in HepG2 cells in response to Utt-B treatment. Student’s t test was used for statistical comparison between different groups. ****P ≤ 0.0001.

Administration of Utt-B in the absence of the chemical inhibitors resulted in 50% cytotoxicity and prominent induction of early apoptosis in HepG2 cells. The cytotoxicity induced by varying concentrations of each of the pharmacological inhibitors was evaluated and the sub-toxic concentration was chosen for the pharmacological inhibition studies (Supplementary Fig. 2). Although, each of the chemical inhibitors induced cytotoxicity and mild apoptosis that was statistically significant, none of the inhibitors decreased the cell viability below 70%. However, administration of Utt-B to cells pre-treated with 1 μM erlotinib (Erlo), drastically decreased the profound cytotoxic and pro-apoptotic activities of Utt-B. The resulting cytotoxicity and apoptosis in the cells treated with the combination of Erlo+Utt-B were comparable to that of cells treated with Erlo alone and was far lesser compared to that of the cells treated with Utt-B alone. These observations confirm that Utt-B-mediated down-regulation of EGFR is responsible for its anti-HCC effects (Fig. 3A, B). Based on the earlier findings of the present study, it is evident that both mTOR and MAPK pathways that operate down-stream of EGFR, have a crucial role in the action of Utt-B. Inhibition of the mTOR pathway using rapamycin (Rap) revealed that the cytotoxic and pro-apoptotic effects produced by 5 nM Rap alone was comparable to Erlo alone, and was far lesser than the effects produced by Utt-B alone in the absence of Rap. Surprisingly, the administration of Utt-B to cells pre-treated with Rap resulted in the enhancement of cytotoxicity and apoptosis. These results clearly demonstrate that mTOR does not play a regulatory role in dictating the anti-HCC potential of Utt-B. However, the down-regulation of mTOR could be a consequence of Utt-B-mediated down-regulation of EGFR, the up-stream regulator of the mTOR pathway (Fig. 3C, D). Further, the MAPK/ERK pathway was inhibited using U0126. The cytotoxic and pro-apoptotic effects produced by 2 μM U0126 alone was comparable to Erlo alone and Rap alone, and was far lesser than the effects produced by Utt-B alone in the absence of U0126. However, when Utt-B was administered to cells pre-treated with 2 μM U0126, the profound cytotoxic and pro-apoptotic activities of Utt-B were diminished, despite the presence of EGFR. The resulting cytotoxicity and apoptosis in the cells treated with the combination of U0126+Utt-B was comparable to that of cells treated with U0126 alone and was far lesser compared to that of the cells treated with Utt-B alone (Fig. 3E, F). Notably, immunoblotting analyses validated the time-dependent down-regulation of p-EGFR (Y1068,1045) and p-ERK1/2 (T202/Y204) and the concomitant down-regulation of SREBP-1 in response to Utt-B treatment (Fig. 3G). Taken together, the current findings confirm that Utt-B-mediated down-regulation of the EGFR/ERK signaling axis and its down-stream target, SREBP-1 contribute to the remarkable anti-cancer effects elicited by the compound, possibly via limiting excessive de novo lipogenesis and aberrant lipid metabolism.

### Uttroside B impedes the development of orthotopic hepatic tumors and induces apoptosis, in vivo

**Fig. 4:**
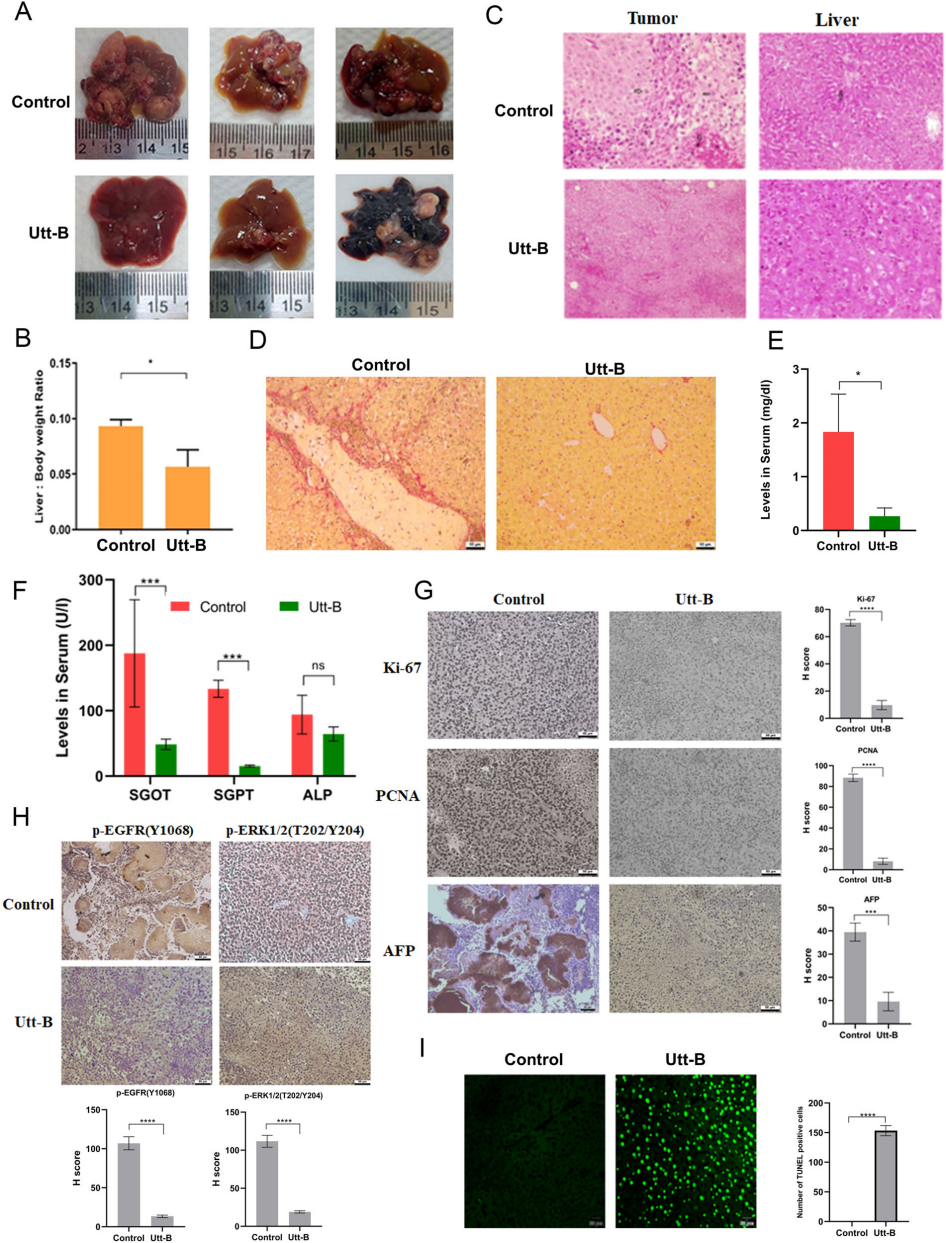
Utt-B hinders the development of hepatic tumors in a murine orthotopic xenograft model of HCC via down-regulation of the EGFR/ERK axis. **A** Representative images of livers bearing orthotopic hepatic tumors in control and Utt-B-treated mice from the orthotopic xenograft model. **B** Graph depicting changes in the liver: body weight ratio in mice from control and treatment groups. Mann-Whitney test was used for statistical comparison between different groups. *P ≤ 0.05. **C** Histopathological analysis of orthotopic tumor and corresponding liver tissues from control group and the tumor-bearing animals from Utt-B treated group of the orthotopic xenograft model. **D** Sirius red staining depicting the excessive deposition of collagen in the livers of control mice and its minimal deposition in livers of mice treated with Utt-B. **E**, **F** Levels of bilirubin and liver enzymes from the control and Utt-B treated groups as assessed by biochemical analysis of serum samples. Student’s t test was used for statistical comparison between different groups. ***P ≤ 0.001; *P ≤ 0.05; ns- non-significant. **G** Immunohistochemical analysis of cancer biomarkers, Ki-67, PCNA and HCC specific biomarker, AFP in the tumor tissues of control and Utt-B treated mice from the orthotopic xenograft model. **H** Immunohistochemical analysis showing the relative tissue expression of p-EGFR and p-ERK1/2 in the tumor tissues of control and Utt-B-treated mice from the orthotopic xenograft model. Student’s t test was used for statistical comparison between different groups. ****P ≤ 0.0001; ***P ≤ 0.001. **I** TUNEL assay showing Utt-B-mediated DNA fragmentation and apoptosis in the livers of mice treated with Utt-B. Student’s t test was used for statistical comparison between different groups. ****P ≤ 0.0001.

In vivo orthotopic xenograft model of HCC which facilitate a better understanding of the therapeutic effects of drugs in the native tumor microenvironment, was established in NSG mice (Supplementary Fig. 1D). Primary solid tumors were observed on the livers of all the untreated animals. Notably, 50% of the animals from the Utt-B-treated group showed normal liver histology, while 20% showed only slight malignant changes in the liver and only 30% of the animals, developed solid hepatic tumors (Fig. 4A). The liver: body weight ratio was much lesser in the treatment group, indicating a positive therapeutic response to Utt-B (Fig. 4B). Histopathological analysis of the tumor tissues and the corresponding liver tissues revealed poorly differentiated grade 4 HCC in the control group animals. Presence of sheets of malignant cells showing mitosis were noted in the hepatic tumor tissues and liver tissues collected from all the control mice (H score: +++ Strong positive). The tumor-bearing mice from the Utt-B group was verified as HCC (H score: ++ Moderate positive) (Fig. 4C). The liver tissues of mice from the Utt-B group which did not develop solid tumors showed binucleate large hepatocytes and signs of dysplasia (H score: + Weak positive). Significant congestion was also observed in the corresponding liver tissues from the control group, while it was mild in that of the animals from Utt-B group (Supplementary Fig. 3A). Excessive collagen deposition associated with the pathogenesis of HCC was noted in the livers of control mice, while it was significantly less in the livers of mice treated with Utt-B, as assessed by Sirius red staining. This observation strongly indicate that Utt-B improves the histopathological features of the livers of mice bearing orthotopic HCC xenografts (Fig. 4D). The elevated levels of liver enzymes such as, SGOT, SGPT, ALP and increased levels of total bilirubin, creatinine and blood urea nitrogen in the serum serve as markers of impaired hepatic and renal dysfunction. The biochemical analysis of the serum indicates a drastic increase in the levels of the liver enzymes, SGOT, SGPT and the levels of total bilirubin, blood urea nitrogen and creatinine in the control mice, while, these levels were stabilized well-within the normal range in the mice treated with Utt-B (Fig. 4E, F; Supplementary Fig. 1E, F). These results suggest that Utt-B stabilizes hepatic and renal function in mice bearing orthotopic HCC xenografts. Immunohistochemical analysis revealed that the expression of the HCC specific biomarker, alpha-foetoprotein (AFP) and the proliferation markers, Ki-67 and PCNA, were significantly high in the control tumor tissue while the expression of all these proteins were markedly reduced in the tumor tissue collected from the only tumor-bearing animals in the Utt-B treatment group, attesting the remarkable efficacy of Utt-B against HCC (Fig. 4G). Furthermore, a drastic reduction in the expression of p-EGFR and p-ERK1/2 was observed in the tumor tissue collected from the tumor-bearing animals in the Utt-B group, compared to the control group (Fig. 4H). Moreover, Utt-B induced drastic DNA fragmentation, a classical hallmark of apoptosis, in the liver tissues of mice bearing orthotopic HCC xenografts, as assessed by TUNEL assay (Fig. 4I). While 50% necrosis was observed in the histopathological analysis of the hepatic tumor tissues collected from the control animals, there were no signs of necrosis in the tumor tissue collected from the ttumor-bearing animals in the Utt-B group. This could be due to the extensive apoptosis induced by Utt-B as evidenced by the results of the TUNEL assay.

### Uttroside B retards the rate of migration, invasion and epithelial-mesenchymal transition (EMT) of HepG2 cells, in vitro

**Fig. 5:**
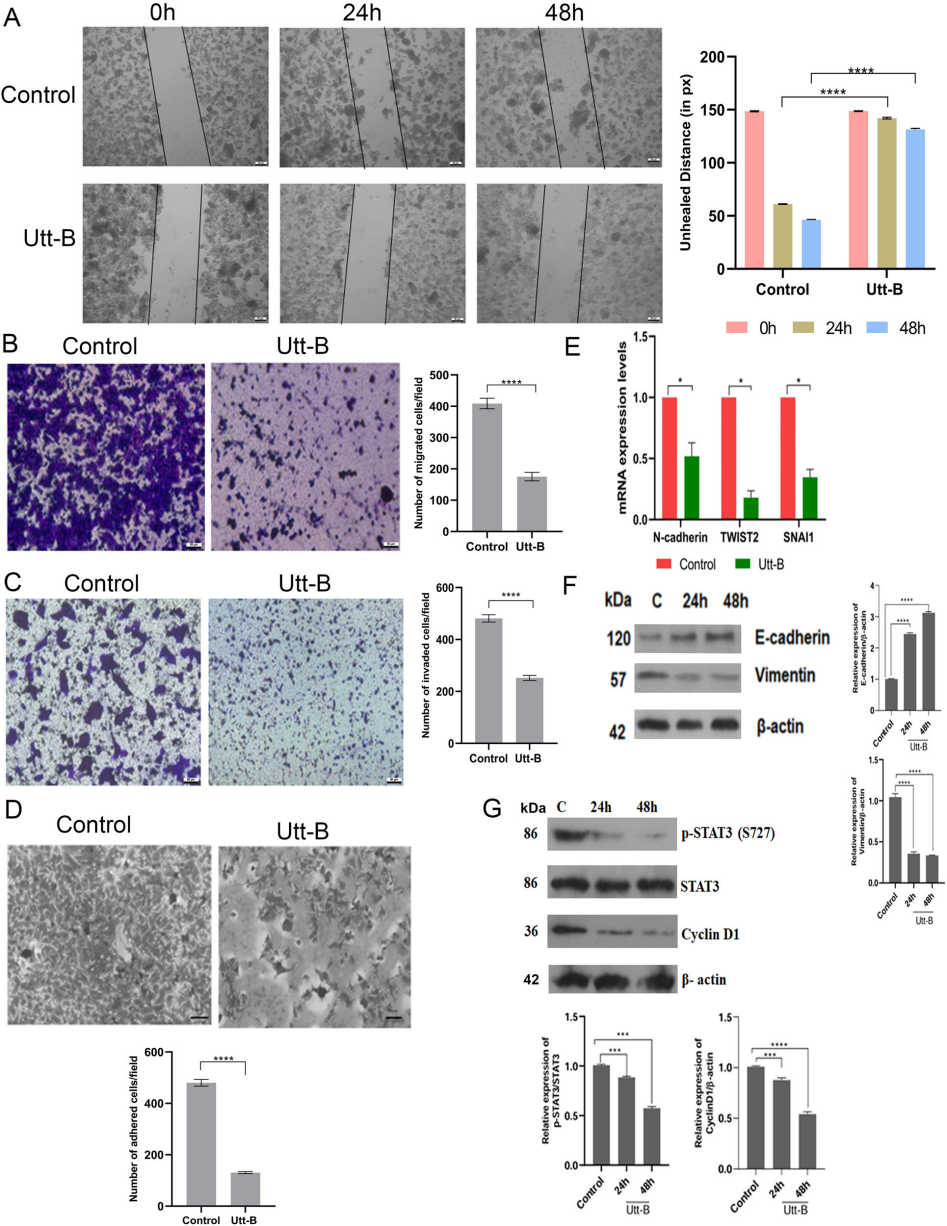
Utt-B significantly inhibits the migration, invasion and adhesion and epithelial-mesenchymal transition of HepG2 cells, via down-regulation of STAT 3 pathway. **A** Wound healing assay depicting the ability of Utt-B in inhibiting the cell migration in treated HepG2 cells as compared to the untreated control cells. Student’s t test was used for statistical comparison between different groups. ****P ≤ 0.0001. **B** Transwell migration assay indicating the anti-migratory effects of Utt-B in HepG2 cells. Student’s t test was used for statistical comparison between different groups. ****P ≤ 0.0001. **C** Transwell invasion assay indicating the anti-invasive properties of Utt-B in HepG2 cells. Student’s t test was used for statistical comparison between different groups. ****P ≤ 0.0001. **D** Cell adhesion assay demonstrating the strong inhibitory effects of Utt-B against the adhesion of HepG2 cells onto Matrigel coated wells. Student’s t test was used for statistical comparison between different groups. ****P ≤ 0.0001. **E** Relative fold change in the mRNA expression levels of EMT markers in HepG2 cells treated with Utt-B as analyzed using qRT-PCR. Student’s t test was used for statistical comparison between different groups. *P ≤ 0.05. **F** Western blot analysis showing the Utt-B-mediated changes in the expression of the EMT markers, E-cadherin and Vimentin in HepG2 cells. **G** Western blot showing the down-regulation of STAT3 pathway in HepG2 cells in response to Utt-B treatment. Student’s t test was used for statistical comparison between different groups. ****P ≤ 0.0001; ***P ≤ 0.001.

Our next attempt was to evaluate the efficacy of Utt-B against invasion and metastasis. The anti-migratory effects of Utt-B was studied using a wound closure assay, where no significant wound closure was observed in the cells treated with 500 nM Utt-B, even after 48 h post-treatment (Fig. 5A). The anti-migratory effects of Utt-B was further confirmed by a cell migration assay, where a drastic reduction in the chemotactic migration was noted in HepG2 cells treated with Utt-B (Fig. 5B). Further, Utt-B led to a marked decrease in the number of cells that penetrate across the ECM barrier and migrate towards the chemoattractant, in the cell invasion assay (Fig. 5C). Interestingly, Utt-B led to a drastic reduction in the number of cells that adhered on to the surface of Matrigel-coated wells in the cell adhesion assay, validating the inhibitory effect of Utt-B on the post-invasion adhesion potential of liver cancer cells, and confirming the anti-metastatic property of Utt-B, in vitro (Fig. 5D). The results of the qRT-PCR revealed that Utt-B decreases the mRNA expression levels of mesenchymal markers such as N-cadherin, TWIST2 and SNAI1 (Fig. 5E). The immunoblotting results indicate that Utt-B induces significant up-regulation of the epithelial marker, E-cadherin and down-regulation of the mesenchymal marker, vimentin, in a time-dependent manner (Fig. 5F). The reduction in the expression of mesenchymal markers and enhancement in the expression of epithelial marker suggests the potential of Utt-B in preventing the epithelial-to-mesenchymal transition, one of the major pre-requisites for the initiation of metastasis. Previous literature suggests the phosphorylation of STAT3 at the S727 residue is commonly mediated by ERK1/2 [19]. Utt-B induces a time-dependent down-regulation of p-STAT3 (S727) and its down-stream target, cyclin D1 (Fig. 5G), suggesting that Utt-B could be preventing the metastasis of HCC, via down-regulation of the EGFR/ERK axis and the subsequent down-regulation of STAT3 pathway. This could be one of the possible mechanisms underlying the strong anti-metastatic potential of Utt-B.

### Uttroside B alleviates the pulmonary metastasis of HCC, in vivo

**Fig. 6:**
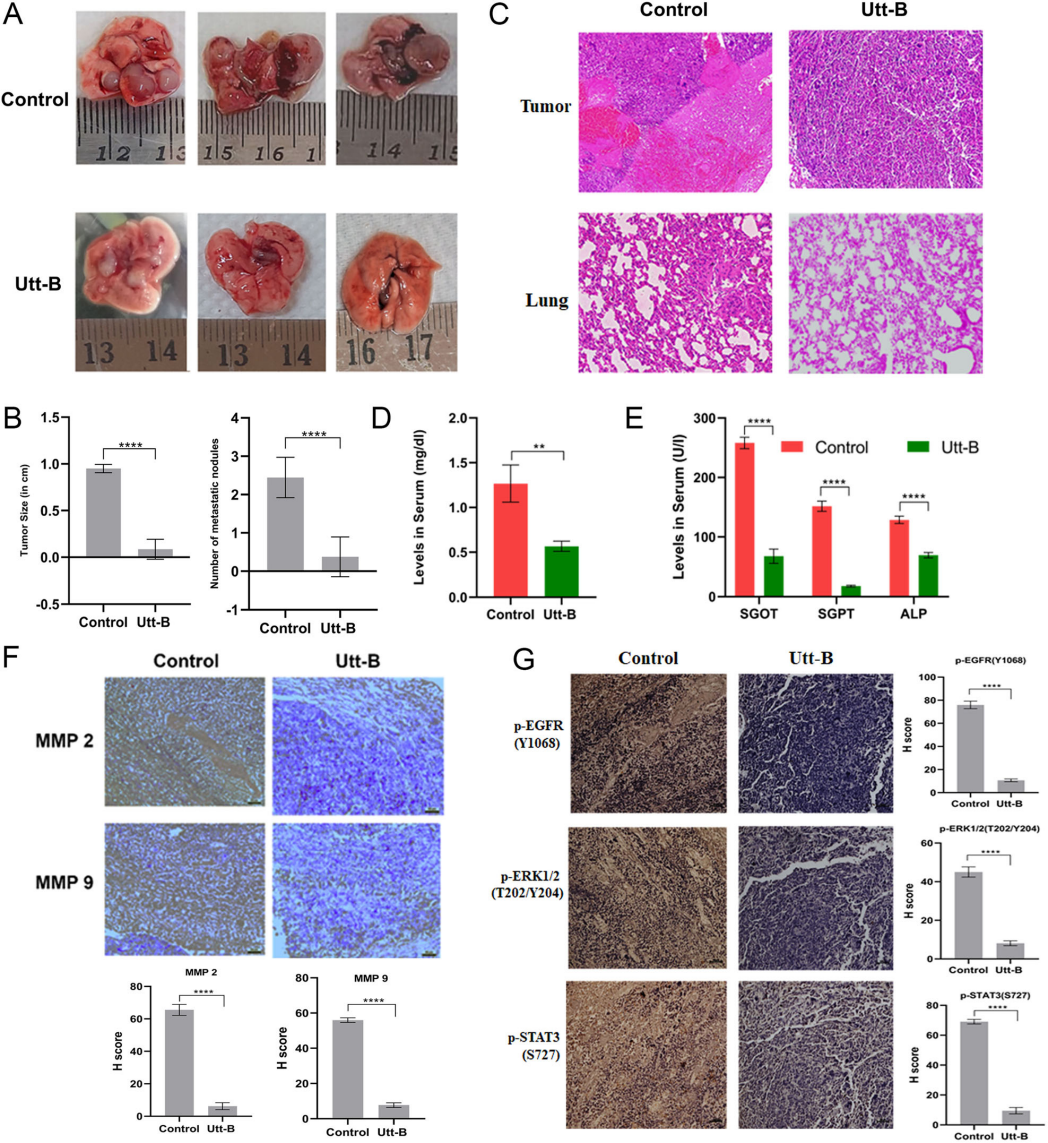
Utt-B mitigates the pulmonary metastasis of HCC, in vivo, via down-regulation of the EGFR/ERK axis and STAT3. **A** Representative images of lungs bearing metastatic tumors in control and Utt-B-treated mice from the murine metastasis model of HCC. **B** Graphical representation of the difference in the size and number of tumor nodules in mice from control and treatment groups. Student’s t test followed by Mann-Whitney test was used for statistical comparison between different groups. ***P ≤ 0.001. **C** Histopathological analysis of the metastatic tumor and corresponding lung tissues of control and Utt-B-treated mice from the murine metastasis model. **D**, **E** Levels of bilirubin and liver enzymes of animals from the control and Utt-B-treated groups as assessed by biochemical analysis of serum samples. Two-way ANOVA was used for statistical comparison between different groups. ****P ≤ 0.0001; **P ≤ 0.01. **F** Immunohistochemical analysis showing the relative tissue expression of EMT markers, MMP-2 and MMP-9 in the metastatic tumors of control and Utt-B- treated mice from the murine metastasis model of HCC. **G** Immunohistochemical analysis showing the relative tissue expression of p EGFR, p-ERK1/2 and p-STAT3 in the metastatic tumors of control and Utt-B-treated mice. Student’s t test analysis was used for statistical comparison between different groups. ****P ≤ 0.0001.

An in vivo murine metastasis model of HCC was established in NSG mice using the invasive human liver cancer cell line, Hep3B (Supplementary Fig. 1G). Solid tumors and large colorless nodules that are indicative of HCC metastasis were developed on the lungs of all untreated animals, while only 30% of the animals from the Utt-B group developed secondary solid tumors on the lungs, while 50% of the animals showed normal lung histology devoid of any malignancy (Fig. 6A). However, 20% of the animals from the treatment group showed signs of malignant changes in the liver. Moreover, the tumor nodules developed on the lungs of animals from the treatment group were much lower in size and number as compared to that of the control mice (Fig. 6B). Histopathological analysis of the metastatic tumors and the corresponding lung tissues validated the occurrence of solid tumors in all the mice from the control group (H score: +++ Strong positive). The tumor nodules isolated from the animals treated with Utt B group were also identified as HCC (H score: ++ Moderate positive). The lungs of control mice showed mild collapse, while that of Utt-B-treated mice showed normal histology. Moreover, the control tumors indicated 50% necrosis whereas, no traces of necrotic changes were visible in the tumor tissue of animals from the Utt-B group (Fig. 6C). Notably, the liver tissues of mice from Utt-B group that did not develop tumors on the lungs showed slight malignant changes (H score: + Weak positive). The malignant changes were also noted in the livers of mice from control group (H score: ++ Moderate positive) (Supplementary Fig. 3B). Tumor necrosis has a positive correlation with intra-tumor hypoxia, vascular invasion and aggressiveness of tumor metastasis [20]. Therefore, the current results confirm that Utt-B drastically decelerates the development of aggressive metastatic tumors in the lungs, in vivo. The levels of SGOT, SGPT, ALP and total bilirubin were stabilized within the normal range in mice treated with Utt-B, while a drastic increase in the levels of these liver enzymes was observed in the control mice (Fig. 6D, E). The renal function parameters such as levels of blood urea nitrogen and creatinine were much lower in the Utt-B-treated mice as compared to that of the control mice (Supplementary Fig. 1H, I). These results suggest that Utt-B stabilizes the hepatic and renal function in mice bearing metastatic HCC tumors. Further, immunohistochemical analysis of the tumor tissues of mice from the control and Utt-B groups revealed a significant reduction in the expression of MMPs, 2 and 9, the molecular markers associated with EMT, in the metastatic tumor tissues, suggesting the therapeutic role of Utt-B in preventing EMT and impeding the metastatic progression of HCC (Fig. 6F). Immunohistochemical analysis demonstrates the elevated expression of p-EGFR, p-ERK1/2 and p-STAT3 in the control tumor tissues. However, there was a marked reduction in the expression of p-EGFR, p-ERK1/2 and p-STAT3 in the tumor tissue collected from the Utt-B group, compared to the control group mice, attesting the possible role of EGFR/ERK axis and STAT3 in regulating the robust anti-metastatic potential of Utt-B (Fig. 6G).

### Silencing of EGFR attenuates the cytotoxic, pro-apoptotic and anti-invasive properties of Utt-B

**Fig. 7:**
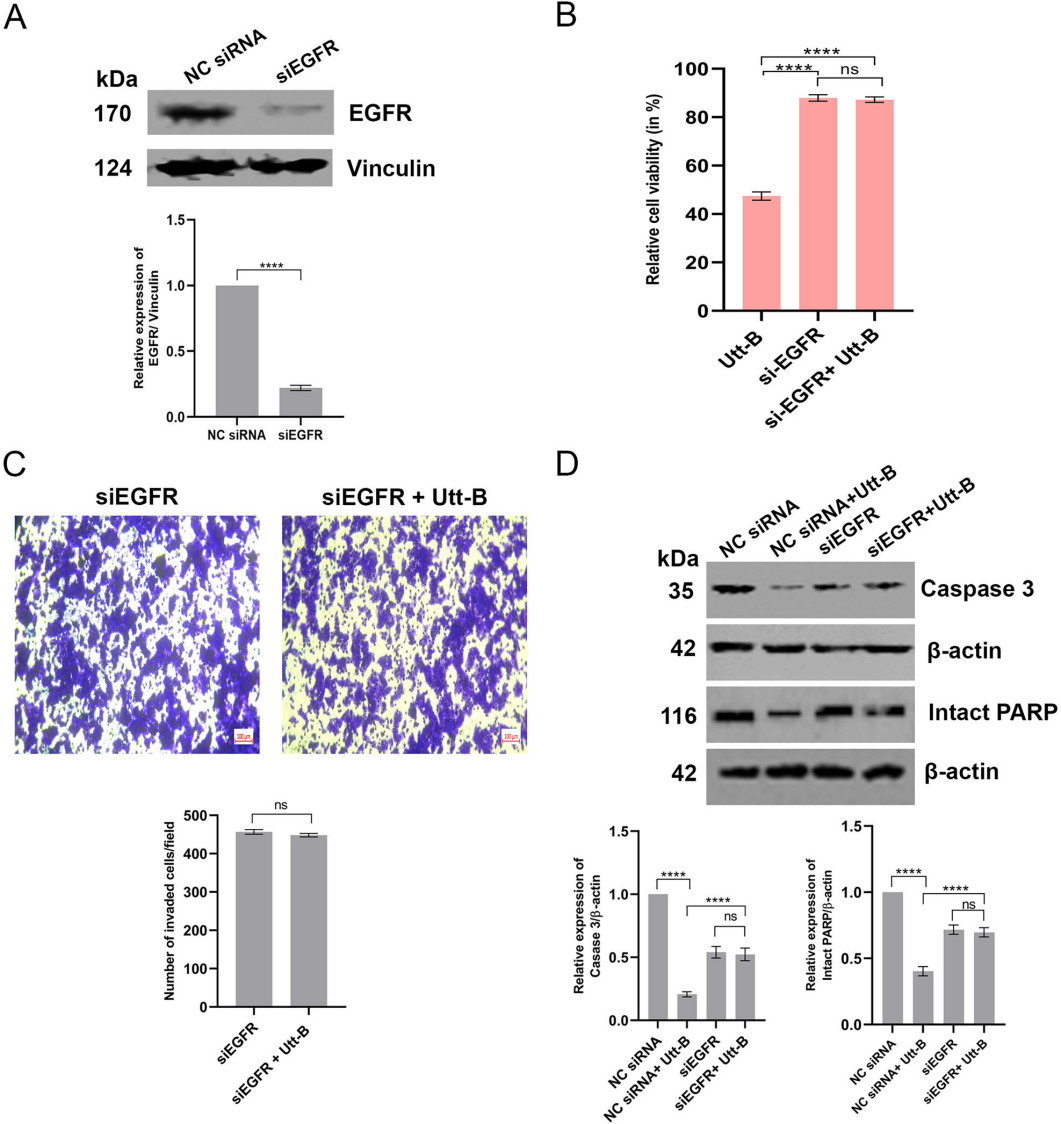
siRNA-mediated EGFR silencing attenuates the cytotoxic, anti-invasive and pro-apoptotic properties of Utt-B. **A** Western blot showing the down-regulation of EGFR in HepG2 cells transfected with Silencer select EGFR siRNA. Student’s t test was used for statistical comparison between different groups. ****P ≤ 0.0001. **B** MTT assay depicting the changes in Utt-B-mediated cytotoxicity in HepG2 cells transfected with NC siRNA or siEGFR. One-way ANOVA followed by Tukey’s post hoc t test analysis was used for statistical comparison between different groups. ****P ≤ 0.0001; ns- non-significant. **C** Transwell invasion assay indicating the effect of Utt-B in HepG2 cells transfected with siEGFR. Student’s t test was used for statistical comparison between different groups. ns- non-significant. **D** Western blot showing the changes in Utt-B-mediated activation of caspase 3 and PARP in HepG2 cells transfected with NC siRNA or siEGFR. Student’s t test was used for statistical comparison between different groups. ****P ≤ 0.0001.; ns- non-significant.

siRNA-mediated silencing of EGFR was performed in HepG2 cells and the partial silencing of EGFR was confirmed by Western blotting (Fig. 7A). Utt-B reduced the cell viability to 50% in HepG2 cells transfected with negative control siRNA (NC siRNA). A slight cytotoxicity was noted in EGFR-silenced control cells (siEGFR). Notably, the viability of EGFR-silenced cells treated with Utt-B (siEGFR + Utt-B) was comparable to that of siEGFR group and much higher than that of Utt-B group (Fig. 7B). Further, it was observed that treatment with Utt-B did not induce any significant retardation in the rate of invasion of cells across the ECM barrier towards the chemoattractant in EGFR-silenced cells (Fig. 7C). Interestingly, Utt-B drastically induced the activation of caspase 3 and PARP in cells transfected with NC siRNA. However, the activation of these key apoptotic markers in EGFR-silenced cells treated with Utt-B (siEGFR + Utt-B) was comparable to that of siEGFR group and much lower than that of Utt-B group (Fig. 7D). Collectively, our results suggest that silencing of EGFR results in the attenuation of the robust cytotoxic, pro-apoptotic and anti-invasive properties of Utt-B and validate EGFR as the key regulator in orchestrating the anti-cancer and anti-metastatic effects of the compound in the context of HCC.

## Discussion

Previous reports by our team have demonstrated the pro-autophagic role of Utt-B via down-regulation of mTOR and MAPK, the key pathways are associated with the induction of autophagy. The transcriptomic and phosphoproteomic analyses in the current study have unraveled EGFR as a potential molecular target of Utt-B. The current study, for the first time, demonstrates the key regulatory role of EGFR, the upstream molecule of MAPK and mTOR, in governing the anti-HCC and anti-metastatic effects of the compound via down-regulation of ERK/SREBP-1 and ERK/STAT3 axes. A recent report by our team has documented that Utt-B gets encapsulated within the lipophilic layer of the extracellular vesicle (EV) membrane, forming complexes with cholesterol molecules [21]. Previous literature suggests that in the resting state, the EGFR organization on the plasma membrane is partly trapped in the cholesterol-containing domains of the plasma membrane [22]. The current in silico findings suggest that Utt-B exhibits a more stable binding with EGFR than mTOR. Therefore, it is possible that Utt-B binds to the extracellular domain of EGFR and induces the endocytosis of the receptor. Previous reports have demonstrated that endocytosis of EGFR impedes its activation by suppressing the autophosphorylation of its tyrosine kinase domains, in turn, leading to the inhibition of the signaling cascade occurring down-stream of EGFR [23]. Several studies have documented the therapeutic effects of EGFR tyrosine kinase inhibitors (TKIs) such as, gefitinib and erlotinib against HCC [24–26]. Kotzka *et al*., have documented ERK and MAPK-mediated activation of SREBP-1 [8, 27]. The elevated expression of SREBP-1 is a characteristic feature of HCC and is responsible for excessive lipid biosynthesis and uptake, which exacerbate the development and progression of HCC [10]. Inhibition of SREBP-1 has yielded promising results in sensitizing HCC to radiofrequency ablation and sorafenib therapy [28, 29]. A previous study suggests that the sustained up-regulation of EGFR and the concomitant STAT3 activation in HCC specimens correlates with the clinical stage of HCC [30]. Furthermore, the study has demonstrated that miR-491 prevents cancer stem cell-like properties and metastasis of HCC cells by blocking the EGFR-mediated activation of STAT3 [30]. A recent study has revealed the role of EMX1-FL in triggering metastasis via activation of EGFR/ERK signaling [31]. Another independent study has demonstrated that afatinib, an EGFR inhibitor, significantly inhibits EMT and tumorigenesis of Huh-7 cells via down-regulation of ERK/VEGF/MMP9 signaling pathway [32]. Notably, the present study is the first report to date, to demonstrate the role of Utt-B in preventing EMT and the pulmonary metastasis of HCC.

Pharmacological safety evaluation of Utt-B, using murine models of acute and sub-chronic toxicity models, conducted by our team have demonstrated that Utt-B stabilizes the hepatic and renal function, and does not induce cardiotoxicity in murine models [13–16]. Further, a physiology-based pharmacokinetic modelling study by our team has provided cues on the pharmacokinetic profile of Utt-B. The study revealed that the half-life of Utt-B is 36.99 h and maximum retention time (MRT) is 53.37 h [21]. However, further in vivo studies may facilitate an in depth understanding of the potential off-target effects of the drug, if any, and aid in substantiating the pharmacokinetic profile in detail. Future studies directed towards studying the influence of Utt-B in modulating metabolic pathways, particularly, the ones associated with lipid biosynthesis, and the potential crosstalk of these pathways with metastatic signalling cascades would be an interesting area of research. Taken together, the findings of the present study elucidate the key role of EGFR in orchestrating the anti-HCC and anti-metastatic effects of Utt-B. The findings of this study attest to the remarkable therapeutic efficacy of Utt-B as a promising drug candidate against primary and metastatic HCC and highlight its potential to be evaluated in the clinics for the benefit of HCC patients having limited prognosis and therapeutic options.

## Materials and Methods

Isolation, purification and characterization of Utt-B was done as previously reported [13].

### Chemicals

Dulbecco’s Modified Eagle Medium (DMEM) (GIBCO, Cat. No. 12800-017), Fetal Bovine Serum (GIBCO Cat. No. 10270-106), streptomycin sulfate (GIBCO, Cat. No. 11860-038), were obtained from Invitrogen Corporation (Grand Island, USA). Poly Excel HRP/DAB detection system universal kit (PathnSitu Biotechnologies Pvt. Ltd, India, Cat. No. OSH001) was used for immunohistochemistry experiments. MTT reagent (Cat. No. D0801) was purchased from TCI Chemicals (India) Pvt. Ltd. Matrigel (Cat. No. 356231) was procured from Corning. Antibodies against β-actin (Cat. No. 12620S), p-EGFR-Tyr1068 (Cat. No. 3777S), EGFR (Cat. No. 2239S), p-ERK1/2-Thr202/Tyr204 (Cat. No. 9101S), ERK1/2 (Cat. No. 9102S), p-mTOR-Ser2481 (Cat. No. 2974S), Caspase 3 (Cat. No. 9662S), Vinculin (Cat. No. 4650S), PARP (Cat. No. 9532S) and p-STAT3-Ser727 (Cat. No. 9136S) were obtained from Cell Signaling Technologies (Beverly, MA, USA) and the antibodies against PCNA (Cat. No. sc25280), Ki67 (Cat. No. sc23900), p-EGFR-Tyr1045 (Cat. No. sc57541), STAT3 (Cat. No. sc8019), SREBP-1 (Cat. No. sc13551), Cyclin D1 (Cat. No. sc8396), E-cadherin (Cat. No. sc8426), MMP2 (Cat. No. sc13594), and MMP9 (Cat. No. sc6840), and Annexin V-FITC/PI apoptosis detection kit (Cat. No. sc4252AK) were purchased from Santa Cruz Biotechnology (Santa Cruz, CA, USA). Antibody against vimentin (Cat. No. MA3 41 745) was purchased from Invitrogen. DeadEnd™ Fluorimetric TUNEL System was procured from Promega (Cat. No. G3250). Silencer Select EGFR siRNA and negative control siRNA were purchased from Thermo Fisher Scientific. Lipofectamine LTX reagent with PLUS (Cat. No. 15338100) was procured from Invitrogen, Thermo Fisher, Scientific. Power SYBR Green PCR master mix (Cat. No. 4367659) and High-capacity cDNA kit (Cat. No. 4368814) were purchased from Applied Biosystems. U0126 (Cat. No. 662005) was purchased from Merck Millipore. MycoSensor qPCR Assay Kit (Cat. No. 302107) was purchased from Agilent Technologies.All other chemicals were purchased from Sigma Chemicals (St. Louis, MO, USA) unless mentioned otherwise.

### Cell Culture

The liver cancer cell lines, HepG2, Huh7 and Hep3B were from obtained from NCCS, Pune, India. All the cell lines were cultured in DMEM supplemented with 10% FBS. All the cell lines used in this study were mycoplasma-tested and were free from mycoplasma contamination.

### Animal experiments

In vivo experiments were conducted in accordance with Institutional Animal Ethical Committee guidelines (IAEC Approval No: IAEC/765/RUBY/2019 and IAEC/944/HARI/2023). Mice were housed in a 12 h light/dark cycle with access to standard food pellets and autoclaved water *ad libitum*. 4-5 weeks old (body weight around 24–28 g) NOD-SCID Gamma (NOD.CB17-Prkdcscid/J Gamma) male mice were used for the experiments. The mice were anaesthetized using ketamine (100 mg/kg body weight) and xylazine (16 mg/kg body weight). An incision was made in the abdominal region. 2×10^6^ Huh 7 cells (dissolved in 1:1 PBS: Matrigel) were orthotopically injected into the left hepatic lobe using a Hamilton syringe. The incision was closed using surgical clips. The mice were monitored until they regained consciousness. The surgical clips were removed after the wound was completely healed after 5-7 days post-surgery. One-week post-cell injection, the mice were randomly assigned to 2 groups (*n* = 9). Group I was treated with PBS alone, Group II received an intraperitoneal injection (IP) of Utt-B dissolved in PBS at a dose of 10 mg/Kg body weight on alternate days. Drug treatment was continued for up to 4 consecutive weeks, after which animals were euthanized and the blood and tissue samples were collected. The HCC metastasis model was established by injecting 1 × 10^6^ Hep3B cells (dissolved in 1:1 PBS: Matrigel) via the lateral tail vein of NSG mice. Two days i.e., 48 h post-injection, the mice were randomly assigned to 2 groups (*n* = 9). Group I received the vehicle, and Group II received an intraperitoneal injection of Utt-B in PBS (10 mg/kg body weight) respectively, on alternate days for 8 consecutive weeks, followed by the euthanasia of animals and subsequent procurement of blood and tissue samples for further analyses. For both the animal experiments, the animals were randomly assigned to control and treatment groups and the administration of PBS/Utt-B and histopathological analyses were performed in a blinded manner.

### MTT assay

MTT assay was performed as previously described [16]. The cells were incubated for 72 h post-treatment with Utt-B, siRNA, Erlotinib, Rapamycin, and U0126. HepG2 cells were pre-treated with the respective inhibitor 6 h prior to the treatment with 500 nM Utt-B for the pharmacological inhibition studies.

### Detection of apoptosis by Annexin V-FITC/PI fluorescence microscopy

Apoptosis was detected using Annexin V-FITC/PI staining followed by fluorescent microscopy as previously described [16]. Curcumin (25 μM) was used as the positive control.

### Immunofluorescence studies

Immunofluorescence studies were performed as previously described [16, 21].

### Wound healing assay

Wound healing assay was performed as previously described [16].

### Boyden chamber cell migration and invasion assay

Migration and invasion assays were performed in 24-well plate with Transwell inserts (8 μm pore size) as previously described [33].

### Cell adhesion assay

Cell adhesion assay was performed using 96-well plates as previously described [33].

### RNA isolation and cDNA synthesis

RNA isolation from cells was performed by TriZol method. cDNA synthesis was done using High-Capacity cDNA synthesis kit (Applied Biosystems).

### Real-Time PCR

Real-Time PCR was performed using Power SYBR Green PCR master mix, Applied Biosystems. GAPDH was used as the housekeeping gene for normalization. The data were calculated using the 2^-∆∆Ct^ method [34]. The details of the primers used in the study have been included in Supplementary Table 1.

### Nanostring nCounter assay

For NanoString analysis, samples were quantified using Qubit RNA HS assay (Invitrogen, Cat # Q32855) kit and qualitatively analysed on Agilent 2100 bioanalyzer nano chip (Agilent, Cat # 5067-1511). Codeset reaction for nCounter Custom Gene Panel (NanoString Technologies) was carried out as per the Manual (nCounter Gene Expression Panel and custom codeset user manual, MAN-10056-06). The Raw data files were downloaded from nCounter SPRINT machine and analyzed using nSolver™ 4.0 (NanoString Technologies) software. The fold change values and unpaired Student’s t test-based p-value were calculated using the ‘calculate ratio’ module from nSolverTM 4.0 Analysis Software (MAN C0019-08). Differentially expressed genes were identified with thresholds of p-value 1.2 (upregulated) or Fold change negative probe counts. All statistical analysis was performed by using R (version 4.0.2, https://www.r-project.org/). p-value less than 0.05 was considered statistically significant.

### Phosphoproteomics analysis

Phosphoproteomic analysis was carried out using metal oxide affinity enrichment coupled with LC-MS/MS as previously described [35].

### siRNA-Transfection

Around 1.5 ×10^4^ HepG2 cells/well were seeded in a 24-well plate and were transfected with negative control or EGFR siRNA for 48 h using Lipofectamine LTX reagent with PLUS procured from Invitrogen, Thermo Fisher, Scientific, according to the manufacturer’s protocol. 150 nM of Silencer Select EGFR siRNA and 200 nM of negative control siRNA were transfected to the cells. Silencer Select EGFR siRNA and negative control siRNA (NC siRNA) were purchased from Thermo Fisher Scientific and were dissolved in siRNA buffers recommended by the manufacturer.

### Western blotting

Western blotting analyses were carried out as previously described [16, 36].

### Measurement of haematological and serum biochemical parameters

Analysis of the liver and renal function profiles of mice was performed using Animal Biochemistry Analyzer (DryChem NX-500; Fuji Film).

### H&E staining

Haematoxylin and Eosin staining was performed as previously described [16].

### Sirius red staining

Sirius Red staining for detecting collagen deposition and fibrotic changes in tissue sections was performed as previously described [37].

### TUNEL assay

TUNEL assay was performed using Dead End Fluorimetric TUNEL System (Promega) following the manufacturer’s instructions.

### Immunohistochemistry

Immunohistochemical analysis was done using the PolyExcel HRP/DAB detection system universal kit according to the manufacturer’s protocol.

### In silico docking studies

In silico docking studies were performed using the program Schrodinger suite (Maestro 10.4). The crystallographic structures were retrieved from Protein Data Bank for the study. The structures were corrected using protein preparation wizard which ensures the structural accuracy of the initial protein structure. Crystallographic waters were removed, missing hydrogens were added and minimization was performed using OPLS3 forcefield. Chemical structure of Utt-B (CID:44566638) was downloaded from PubChem and prepared using the Ligprep module. Receptor grid was generated around the co-crystallized ligand with the receptor grid generation module. Molecular docking studies were performed using Glide in standard precision (SP) mode.

### Statistical analysis

Data represents the results for experiments performed in triplicate. The quantification of Western blots and immunohistochemistry images were carried out using Image J software. The statistical analysis was performed using Graph Pad Prism software (Graph Pad Software Inc., San Diego, CA, USA). Statistical tests used were Student’s t test, One way ANOVA, Two-way ANOVA. The error bars represent ± SD, taken from three independent experiments.

## Supplementary information

Supplementary data and original blots

Supplementary Table

## Data Availability

Raw data compliant with the institutional confidentiality policies are available upon request. Data requests should be sent to the corresponding author.
