# Supplementary material for: Uttroside B, a US FDA-designated ‘Orphan Drug’, mitigates the development of hepatocellular carcinoma and its pulmonary metastasis via EGFR/ERK-mediated inhibition of SREBP-1 and STAT-3
Source: Cell Death Discov. 2026 Apr 16;12:250. doi: 10.1038/s41420-026-03055-5 (PMC13201800; doi:10.1038/s41420-026-03055-5)
Supplement: Supplementary file 1 — Supplementary data and original blots [file 41420_2026_3055_MOESM1_ESM.pdf]

Supplementary Figure 1

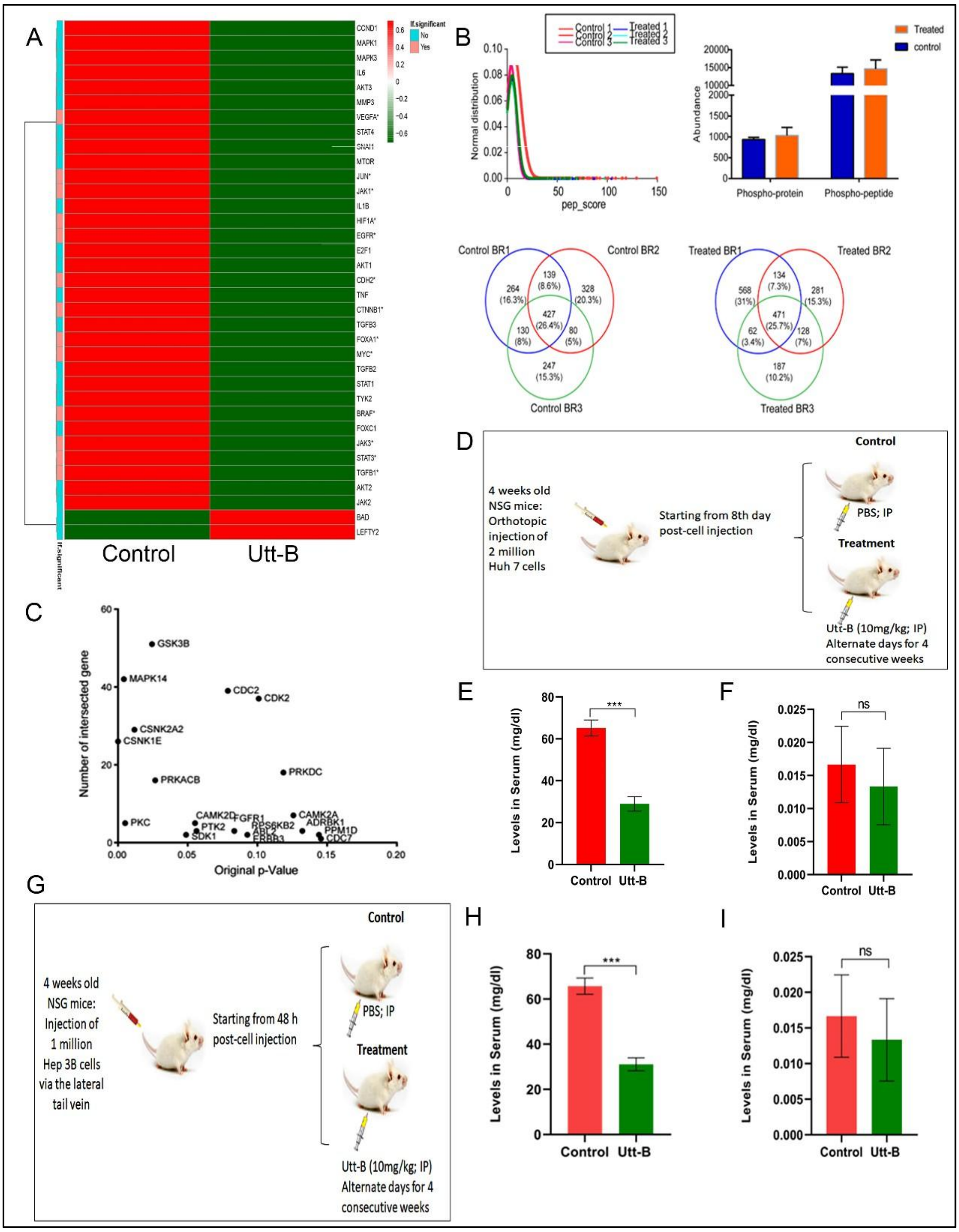

Supplementary Figure 1: Utt-B down-regulates major survival pathways in HepG2 cells and stabilizes the renal function in murine models of primary and metastatic HCC. (A) Representative heat map of the differentially regulated genes in Utt-B-treated HepG2 cells with respect to the control and graph showing commonly enriched kinases in both control and Utt-B groups. **(B)** QC data of phosphoproteomics analysis and data depicting the number of differentially expressed phosphoproteins in the control and Utt-B-treated HepG2 cells in three

biological replicates. **(C)** Kinome extraction data showing the enriched kinases common to both control and Utt-B treatment groups. **(D)** Schematic representation of the experimental design for the orthotopic xenograft model of HCC, *in vivo*. **(E-F)** Renal function profile of animals from the control and Utt-B treated groups from the orthotopic xenograft model of HCC, as assessed by biochemical analysis of serum samples. Student's t test analysis was used for statistical comparison between different groups. ***P ≤ 0.001; ns- non-significant.

(G) Schematic representation of the experimental design for the murine metastasis model of HCC, *in vivo*. **(H-I)** Renal function profile of animals from the control and Utt-B-treated groups from the murine metastasis model of HCC, as assessed by biochemical analysis of serum samples. Student's t test analysis was used for statistical comparison between different groups. ***P ≤ 0.001; ns- non-significant.

Supplementary Figure 2

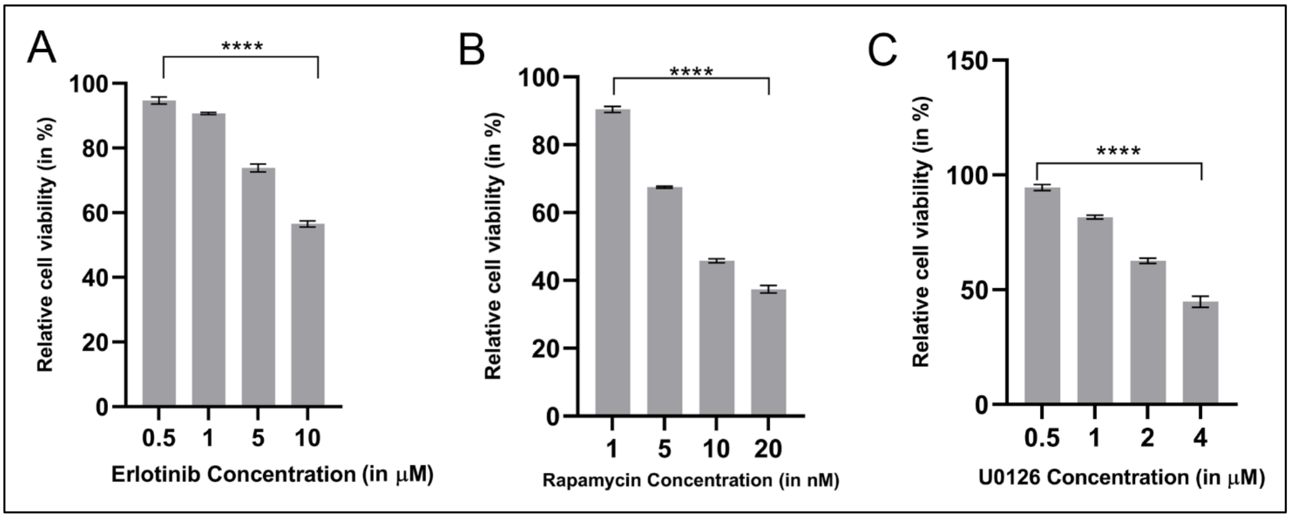

Supplementary Figure 2: Dose dependent cytotoxicity of pharmacological inhibitors in HepG2 cells. Cytotoxicity analysis of **A)** Erlotinib **B)** Rapamycin and **C)** U0126. One-way ANOVA was used for statistical comparison between different groups. ****P ≤ 0.0001.

Supplementary Figure 3

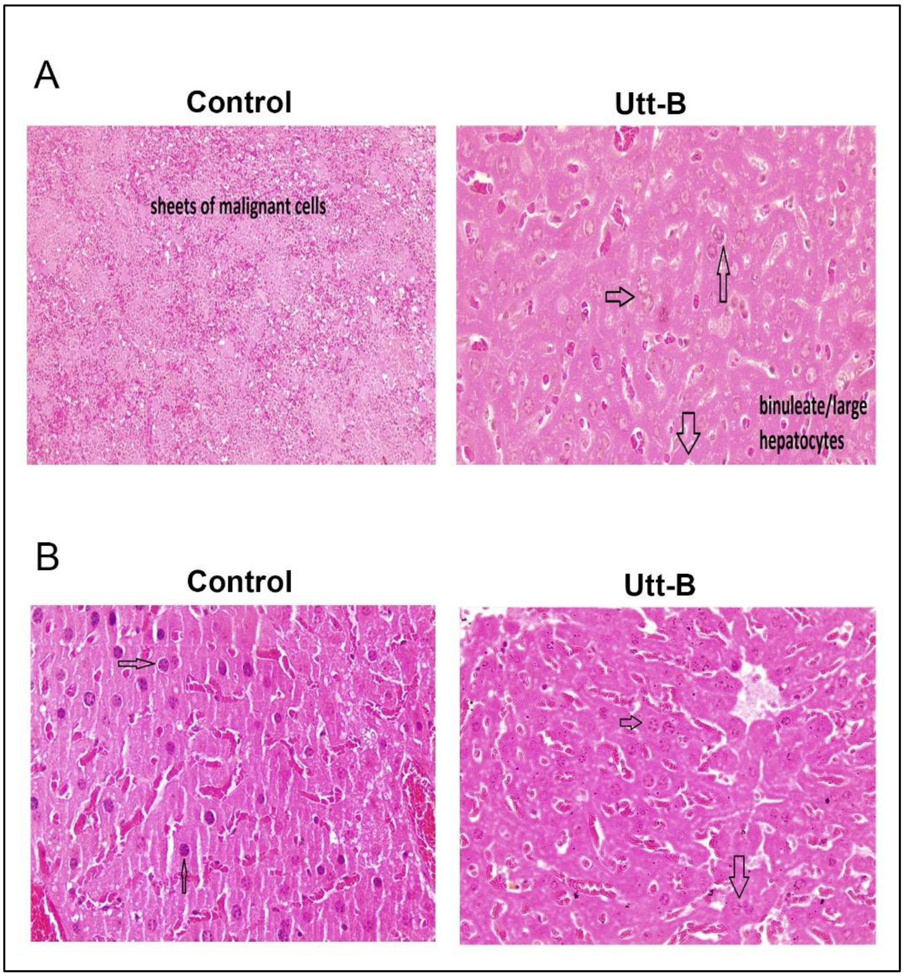

Supplementary Figure 3: Histopathological analysis of livers of mice in orthotopic tumor xenograft model and murine metastasis model. (A) Representative histopathological images of liver tissues of mice from orthotopic xenograft model showing malignant changes **B)** Representative histopathological images of liver tissues of mice from metastasis model showing malignant changes.
