# Supplementary material for: Uttroside B, a US FDA-designated ‘Orphan Drug’, mitigates the development of hepatocellular carcinoma and its pulmonary metastasis via EGFR/ERK-mediated inhibition of SREBP-1 and STAT-3
Source: Cell Death Discov. 2026 Apr 16;12:250. doi: 10.1038/s41420-026-03055-5 (PMC13201800; doi:10.1038/s41420-026-03055-5)
Supplement: Supplementary file 2 — Supplementary Table [file 41420_2026_3055_MOESM2_ESM.doc]

**Supplementary Table 1**

| **Gene** | **Primer Sequence** | | **Annealing Temperature (°C)** |
| --- | --- | --- | --- |
| N-cadherin | Forward | GCAGATAGCCCGGTTTCATTTGAG | 63.0 |
|  | Reverse | AGGGCATTGGGAATCGTCAGCA | 63.0 |
| TWIST 2 | Forward | TCTGACAAAAGCTGAGCAAGATCC | 60.3 |
|  | Reverse | CTGCAGCTGGTCATCTTATTGTC | 60.6 |
| SNAI 1 | Forward | AATCCAGAGTTTACCTTCCAGCAGC | 61.0 |
|  | Reverse | AGCCTTTCCCACTGTCCTCATCT | 61.0 |
| GAPDH | Forward | AGGCAACTAGGATGGTGTGG | 63.9 |
|  | Reverse | TTGATTTTGGAGGGATCTCG | 63.8 |

**Supplementary Table 1: Details of human primers used in the study.**
